# Comparing T Cell Subsets in Broncho-Alveolar Lavage (BAL) and Peripheral Blood in Patients with Advanced Lung Cancer

**DOI:** 10.3390/cells11203226

**Published:** 2022-10-14

**Authors:** Annapaola Mariniello, Fabrizio Tabbò, Davide Indellicati, Martina Tesauro, Nicole Alessia Rezmives, Maria Lucia Reale, Angela Listì, Enrica Capelletto, Simona Carnio, Valentina Bertaglia, Caterina Mecca, Lorena Consito, Marco De Filippis, Maristella Bungaro, Chiara Paratore, Massimo Di Maio, Francesco Passiglia, Luisella Righi, Dario Sangiolo, Silvia Novello, Massimo Geuna, Paolo Bironzo

**Affiliations:** 1 Department of Oncology, University of Torino at San Luigi Gonzaga Hospital, 10043 Orbassano, Italy; 2 Department of Immunology and Microbiology, Emory University School of Medicine, Atlanta, GA 30322, USA; 3 Immuno-Pathology Unit, Mauriziano Umberto I Hospital, 10128 Torino, Italy; 4 Department of Oncology, University of Torino at Mauriziano Umbero I Hospital, 10128 Torino, Italy; 5 Laboratory of Experimental Cell Therapy Candiolo Cancer Institute FPO—IRCCS, 10060 Torino, Italy

**Keywords:** lung cancer, BAL, immunology, T cell, PD-1

## Abstract

Background: Lung cancer (LC) tissue for immunological characterization is often scarce. We explored and compared T cell characteristics between broncho-alveolar lavage from tumor affected (t-BAL) and contralateral lung (cl-BAL), with matched peripheral blood (PB). Methods: BAL and PB were collected during bronchoscopy for diagnostic and/or therapeutic purposes in patients with monolateral primary lesion. Results: Of 33 patients undergoing BAL and PB sampling, 21 had histologically-confirmed LC. Most cases were locally-advanced or metastatic non-small cell LC. T cell characteristics were not significantly different in t-BAL vs. cl-BAL. Compared to PB, CD8 T cells in BAL presented features of immune activation and exhaustion (high PD-1, low IFN-g production). Accordingly, regulatory CD4 T cells were also higher in BAL vs. PB. When dichotomizing T cell density in t-BAL in high and low, we found that PD-L1 expression in LC was associated with T cell density in t-BAL. T-BAL with high T cell density had higher %IFN-g+CD8 T cells and lower %T-regs. Conclusion: In BAL from advanced LC patients, T cells present features of exhaustion. T cells in t-BAL could be the best surrogate of tumor-infiltrating T cell, and future studies should evaluate T cell phenotype and density as potential biomarkers for cancer immunotherapy outcome.

## 1. Introduction

Despite the positive results of low dose CT screening programs, lung cancer is still diagnosed largely at advanced stages, when surgical resection and a definite cure are mostly unfeasible [1,2,3]. This not only explains the overall poor prognosis but also implies that diagnosis is mostly made on small core biopsies, with a limited tissue amount to perform deep molecular and histological characterization. 

Tissue availability represents a well-acknowledged challenge in non-small cell lung cancer (NSCLC) routine clinical practice, where the role of baseline biomarkers’ identification has become essential to guide treatment decision [4]. Indeed, the treatment landscape for NSCLC has been enriched with highly effective strategies, with immunotherapy being the mainstay for non-oncogene addicted patients [4,5]. Monoclonal antibodies targeting the co-inhibitor T cell receptors Programmed Death-1 (PD-1) or its ligand (PD-L1)—and, more recently, Cytotoxic T Lymphocyte Antigen-4 (CTLA-4)—have proven successful in terms of survival benefit, and are now widely used as monotherapy or in combination [6].For treatment of LC, PD-L1 expression assessed with immunohistochemistry using the tumor proportion score (TPS), is the only licensed predictive biomarker of response to checkpoint inhibitors available so far—although encumbered by several limitations [7,8]. The search for alternative response and toxicity biomarkers has brought to attention that blood may represent a valuable source for biomarker discovery and testing besides tumor tissue. Non-invasive and easy to assess, peripheral blood (PB) sampling allows for longitudinal monitoring of certain biomarkers of interest along the treatment course [7,9,10].

Broncho-alveolar lavage (BAL) represents a minimally invasive procedure consisting of the collection of instilled sterile saline solution into a subsegment of the lung. BAL can be obtained during fiber-bronchoscopy, which represents the technique of choice for biopsy or re-biopsy in all cases of large, central lesions [4]. Traditionally, BAL has been used for microbiologic analysis or immune cell phenotyping for the differential diagnosis of a variety of lung conditions, ranging from hypersensitivity pneumonia to interstitial lung disease [11]. More recently, given the limited tissue availability for LC at advanced stages, BAL has raised interest as a potential tool to dissect the biology of LC and of its immune microenvironment, with potential prognostic and predictive implications on treatment outcome [12,13,14]. Here, in BAL performed during diagnostic or therapeutic fiber-bronchoscopy, we aimed to describe and compare the T cell characteristics between BAL from the tumor-affected lung (t-BAL) with BAL from contralateral lung (c-BAL) and PB. We also explored the clinico-pathological associates of T cell density in t-BAL. 

## 2. Methods

### 2.1. Study Design and Population

This prospective observational study had an exploratory design, with the primary aim of comparing T cell populations in t-BAL, cl-BAL, and PB. Retrospectively, we focused on T cell density in t-BAL, using the median value of T cell frequency (of total cells in t-BAL) to discriminate t-BAL samples with low T cell density from those with high T cell density. We searched for associations with clinico-pathological characteristics and described the immune features of t-BAL samples with low vs. high T cell density.

We considered consecutive patients who underwent fiber-bronchoscopy during routine diagnostic work-up, in case of lung lesion(s) suspicious of primary tumor or in case of histologically-confirmed LC re-biopsy. Patients with histologically-confirmed LC undergoing fiber-bronchoscopy for therapeutic purposes (e.g., disobstructive bronchial laser) were also included. Screening and fiber-bronchoscopy planning of candidate patients was performed by the Thoracic Oncology Unit and/or the Interventional Pneumology Unit of the San Luigi Gonzaga University Hospital (Orbassano, Italy).

Key inclusion criteria were the presence of monolateral lung lesion and the ability to sign an informed consent. Patients presenting with severe respiratory failure and severe comorbidities, in whom BAL could worsen residual lung functionality, were excluded.

At the time of fiber-bronchoscopy, PB and t-BAL and cl-BAL were collected and analyzed. After the procedures above, patients were followed up for clinical characteristics in an anonymized database. Whenever fiber-bronchoscopy was performed for diagnostic purposes, only patients with primary LC were included (non-small or small cell histologies).

The collected clinico-pathological data included histological features, molecular profile, PD-L1 expression on tumor cells, 8th TNM stage [15], smoking status, comorbidities, and active oncological treatments with the relative tumor response according to the RECIST criteria [16].

### 2.2. Bronchoalveolar Lavage Fluid and PB Collection

All patients underwent BAL sampling during diagnostic/therapeutic fiber-bronchoscopy at the Interventional Pneumology Unit of the San Luigi Gonzaga University Hospital (Orbassano, Torino, Italy). After intravenous sedation (midazolam 2/2.5 mg, fentanyl 50 mcg) bilateral BAL was performed by instilling a 0.9% saline solution 60 mL in both the lung harboring the suspicious/confirmed tumor and the contralateral one. After recovery, bloody BAL fluids and recovered BAL fluids <30% were excluded from the analyses. PB samples were collected at the time of fiber-bronchoscopy, with matched BAL samples.

### 2.3. Samples Processing and Flow Cytometry Analysis

After collection, BAL and PB samples were processed at the Immuno-Pathology Unit of the Ordine Mauriziano Hospital (Torino, Italy).

BAL lymphocyte immunophenotyping and T cell subsetting were performed by staining for flow cytometry with the antibodies described in Table 1. 

FoxP3+ T regulatory cells were identified using a surface/intracellular staining method (FoxP3 Intracellular fix and perm staining set—eBioscience™, Thermo Fisher, Italy). Briefly, 100 μL of cell suspension was stained for surface markers with mixture 4 (Table 1), incubated 15 min, washed once with PBS/BSA, resuspended in a fixation buffer and then treated following the manufacturer’s instruction. Ten μL of Pacific Blue (PB) conjugated anti FoxP3 antibody (eBioscience) were used for Foxp3 detection.

Ex vivo stimulation of T cells with PMA/Ionomycin/Brefeldin was used to identify interferon-gamma (IFN-g) producing CD8 T cells, and T helper 1 (Th1), T helper 2 (Th2), and T helper 17 (Th17) CD4 T cells based on the production of IFN-g, IL-4, and IL-17, respectively [17]. The inherent intracellular cytokine analysis was performed as follows: 250 μL of cell suspension were incubated with PMA/Ionomycin/Brefeldin for 4 h at 37 °C in RPMI/AB, then washed twice with RPMI/AB. The cell pellet was resuspended in 100 μL of the same medium and labeled with the antibody mixture 5 (Table 1). After 15 min, 100 μL of fixative (IntraPrep kit—Beckman Coulter, Italy) were added, incubated for 15 min, washed once with PBS/BSA, then resuspended in 100 μL of the permeabilizing solution. Five μL each of PB anti-IL17, FITC anti-IFNγ, and PC7 anti-IL4, were added. After 30 min of incubation, samples were washed once with PBS/BSA, resuspended in 500 μL of the same medium, and immediately acquired with flow cytometry.

PB basic immunophenotype was performed using a lyse no-wash technique with a single platform absolute count (Flow Count beads, Beckman Coulter). The remaining PB sample was centrifuged on density gradient (Lymphoprep, StemCell Technologies, Voden Medical Instruments, Italy) to isolate mononuclear cell. T-cell subsetting, Tregs analysis, and intracellular cytokine analysis for the identification of Th1, Th2, and Th17 CD4+ T-cells were performed as previously described for BAL samples.

All samples were acquired with a 3-lasers 10-colors Navios cytometer and Navios software (Beckman Coulter) collecting at least 100–500,000 total events. Analysis of the lymphocyte subpopulations was performed using Kaluza software ver. 2.1 (Beckman Coulter) using a sequential gate strategy: doublet exclusion, debris/unlysed erythrocytes exclusion, CD45/SSC or CD3/SSC and FSC/SSC lymphocyte identification.

### 2.4. Tumor Specimens

Diagnosis of LC was performed by an experienced pathologist and histological subtypes were established based on morphological and immunohistochemical staining (CgA, Synaptophysin, CD56, NSE, Ki67, TTF-1, CK7, p40/63). When missing (as in cases of SCLC), PD-L1 TPS was assessed retrospectively. In all the cases, for PD-L1% TPS, the Dako 22C3 immunohistochemistry assay was used.

### 2.5. Statistics

Summary graphs and statistics were performed in GraphPad Prism v9. FACS data from t-BAL were compared with cl-BAL and with peripheral PB in separate analyses using two-tailed T-test or Mann–Whitney test. For correlation analyses, Pearson’s test was used, and, to explore the associations between T cell density and clinical variables, Chi-square test was used. Two-tailed *p* < 0.05 was considered statistically significant. No correction for multiplicity was applied.

## 3. Results

### 3.1. Patients’ Characteristics

From October 2018 to February 2020, 33 patients underwent BAL and PB sampling. Of those, 21 had histologically-confirmed LC (11 squamous, 6 adenocarcinoma, 4 small cell LC). Of the remaining 12 cases, 4 were diagnosed with other types of malignancies (renal cell carcinoma, synovial sarcoma, NUT carcinoma, gastric cancer), 4 were lost, 2 patients presented histological samples without tumor cells and deceased before repeating the biopsy, and 1 did not receive an oncological diagnosis but was affected by bacterial pneumonia.

Across the 21 LC patients included in the analysis, the median age was 73 years (range 45–80) at the time of BAL, and 19% were female. The median follow up time after BAL was 2.3 years, with 15 out of 21 patients being deceased at the time of data analysis. Survival data were available for 19 out of 21 patients. Median survival time after BAL was 4.2 months (interquartile range (IQR) 2.2–24.9 months), median survival time after diagnosis was 11.3 months (IQR 2.2–26.3 months), with 5 out of 19 (26.3%) patients still alive as of October 2021.

Detailed clinicopathological characteristics of the study population are shown in Table 2. At the time of BAL, most patients presented with locally-advanced or metastatic disease (19 out of 21). Overall, PD-L1 was available in 20 cases, with a median tumor proportion score of 0.5% (IQR 0–25). The median PD-L1 tumor proportion score was 1% (IQR 0–33.7%) in NSCLC biopsy tissue (16 out of 17), whereas all 4 cases of SCLC were PD-L1 negative. Two out of five adenocarcinoma patients presented Epidermal Growth Factor Receptor (EGFR) mutation: one on exon 21, and the other on exon 20. The remaining patients had non-oncogene addicted disease.

Fiber-bronchoscopy was performed for diagnostic purposes in 17 of the 21 cases (80.9%), while in 2 (9.5%) fiber-bronchoscopy had therapeutic-exploratory intent. Two patients (9.5%) underwent fiber-bronchoscopy for re-biopsy after treatment failure or disease recurrence.

After BAL, the majority (19 out of 21) received active treatment, of whom 2 out of 19 received surgery, and 4 out of 19 received immunotherapy—alone or as consolidation after chemo-RT. At the time of BAL, 4 out of 21 patients had received previous treatment for LC: 3 received radical surgery (of whom 2 were followed by adjuvant chemotherapy) and 1 received sequential chemo-radiotherapy.

Regarding relevant immunological comorbidities, one patients presented with latent HBV+ chronic infection and a previous history of mycobacterial tuberculosis (QuantiFERON + test), one with inactive HBV+ cirrhotic hepatitis, and another patient with radiological sequelae of mycobacterial tuberculosis, in absence of QuantiFERON testing. Five out of 21 patients (23.8%) were receiving low dose oral prednisone (≤10 mg/die), and 1 patient was receiving a high-medium dose of dexamethasone (8 mg/die).

### 3.2. BAL and PB Lymphocytes Profiling

**Figure 1:**
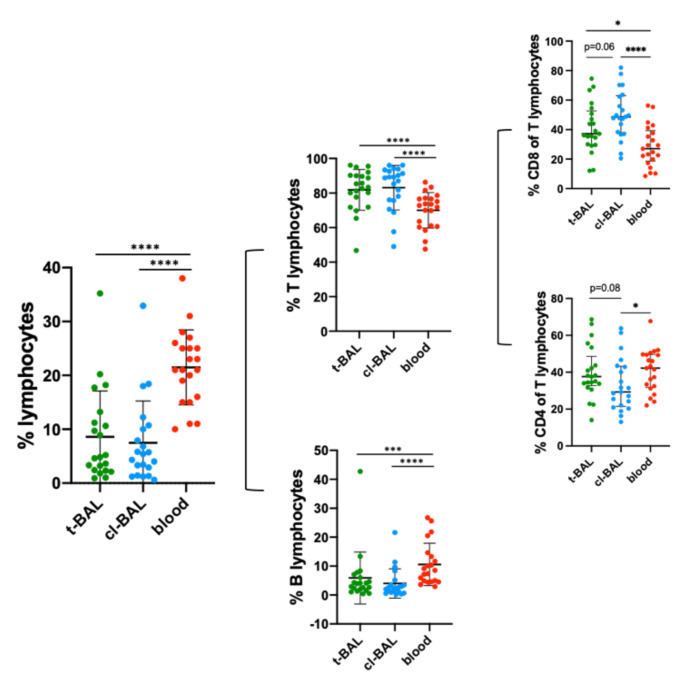
Dot plot showing the lymphocytes frequency of CD45+ cells and relative B and T populations in t-BAL, cl-BAL, and PB of the cohort (21 patients). Mann–Whitney test was performed to detect statistically significant differences across the groups. NS: non-significant. *: *p* < 0.05; ***: *p* < 0.001; ****: *p* < 0.0001.

The total number of BAL and PB samples were 63. The mean cell recovery of t-BAL samples was 5.5 × 10^6^ (SD ± 6.7 × 10^6^), of cl-BAL was 3.5 x10^6^ (SD ± 3.8 × 10^6^) and of PB was 16.4 × 10^6^ (SD ± 15 × 10^6^). In Figures S1 and S2, the gating strategy for lymphocytes in BAL and PB is shown. The relative median frequency of lymphocyte populations in t-BAL, cl-BAL, and PB are shown in Table S1. The median frequency of lymphocytes (of CD45+ SSC ^low^ cells) in t-BAL was 4.8%, cl-BAL 5.4%, and PB 21%, Figure 1). B cells (median t-BAL 3.9%, cl-BAL 2.3%, PB 8.55, t-BAL vs. PB *p* = 0.0005, in Figure 1) and NK cells (median t-BAL 5.8%, cl-BAL 3.2%, PB 15.7%, t-BAL vs. PB *p* = 0.0005) were more abundant in PB, however T cells were higher in both t-BAL (median 83.5%) and cl-BAL (88.8%), compared to PB (72.8%, vs. t-BAL *p* = 0.0008), as shown in Figure 1.

When comparing t-BAL with cl-BAL, t-BAL showed higher CD4 T cells (median 37.6% vs. 29.3%, *p* = 0.08) and lower CD8 T cells frequencies (median 37.2% in cl-BAL vs. 48.6% in t-BAL, *p* = 0.06), in both cases with borderline significance (Figure 1). In PB, the frequency of CD4 T cells was not significantly different from t-BAL (median 42.2%), but CD8 T cells were significantly lower (median 27%) compared to t-BAL (*p* = 0.010) and cl-BAL (*p* < 0.0001).

### 3.3. CD8 T Cells Subsets

Compared to PB, both t-BAL and cl-BAL displayed polarized effector functions of CD8 T cells. The relative frequency of CD8 T cell subsets is shown in Table S2.

**Figure 2:**
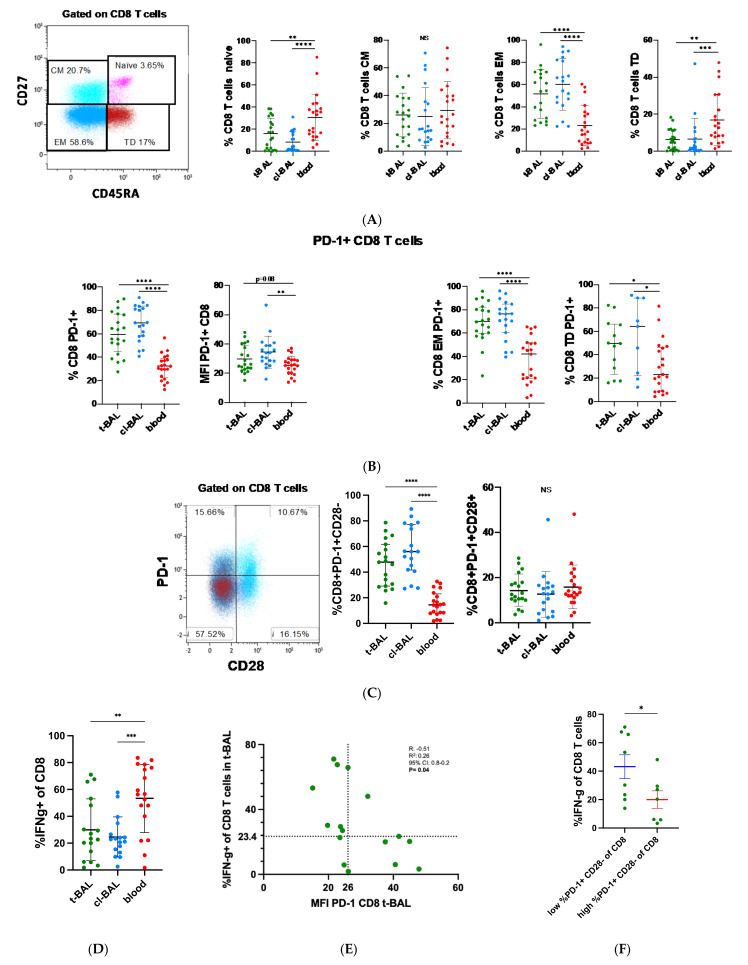
(**A**) FACS plot from the t-BAL of a patient showing naïve, central memory (CM), effector memory (EM), and terminally differentiated (TD) CD8 T cell subsets. The dot plots show the frequencies of these subsets on total CD8 T cells in t-BAL, cl-BAL, and PB of the cohort (21 patients). (**B**) Dot plots showing, from left to right, the frequency of PD-1^+^CD8 T cells on total CD8 T cells, mean fluorescence intensity (MFI) of PD-1 on CD8 T cells, frequency of PD-1^+^ EM C8 T cells on total EM CD8 T cells, and frequency of PD-1^+^ TD C8 T cells on total TD CD8 T cells. (**C**) FACS plot from the t-BAL of a patient showing CD8 populations based on the expression on CD279 (PD-1) and CD28. Dot plots showing frequencies of exhausted (PD-1+CD28-) and early exhausted (PD-1^+^CD28^+^) CD8 T cell populations. (**D**) Dot plot showing frequency of interferon-gamma (IFN-g) producing CD8 T cells (of total CD8 T cells), across t-BAL, cl-BAL and PB assessed with intracellular cytokine staining with PMA/Ionomycin/Brefeldin. (**E**) Pearson’s correlation test between %IFNg+ (of) CD8 T cells and mean fluorescence intensity (MFI) of PD-1 on CD8 T cells. Dashed lines represent the median values of %IFN-g producing CD8 T cells (y axis) and of PD-1 MFI on CD8 T cells (x axis). (**F**) Dot plot showing %IFN-g CD8 T cells in t-BAL with low and high %PD-1+CD28- of CD8 T cells, discriminated based on median % (47.8 IQR 29–62). In (**A**–**D**) Mann–Whitney test was performed to detect statistically significant differences across the groups, except for MFI, where T-test was used. In (**E**), the data were analyzed using T-test. NS: non-significant. *: *p* < 0.05; **: *p* < 0.01; ***: *p* < 0.001; ****: *p* < 0.0001.

As shown in Figure 2A, by using the CD27 and CD45RA markers, we identified in BAL and PB the memory and effector CD8 T cells subsets. In PB, the majority of CD8 T cells more often presented a naïve (median 33.3% vs. 14.7% in t-BAL, *p* = 0.01) or central memory (CM) phenotype (median 24.9% vs. 26.4% in t-BAL, *p* = 0.7), whereas over half of the CD8 T cells from t-BAL and cl-BAL were effector memory (EM) (median 54.1% and 63.1% vs. 17.5 in PB, *p* < 0.0001), without significant difference among them (*p* = 0.3). Of note, terminally differentiated (TD) CD8 T cells were present at low rates in t-and cl-BAL (median 5.1% and 1.6%, respectively *p* = 0.4) and were significantly higher in PB (median 12.7%, vs. t-BAL *p* = 0.003). 

The higher frequency of EM CD8 T cells in cl-BAL and t-BAL remained significant compared to PB even when considering the groups of NSCLC type (cl-BAL and t-BAL vs. PB *p* < 0.0001, t-BAL vs. cl-BAL *p* = 0.5) and squamous cell carcinoma alone (t-BAL vs. PB *p* = 0.002; cl-BAL vs. PB *p* = 0.01, t-BAL vs. cl-BAL *p* = 0.6), which was the most common histological subtype (see Figure S3A,B).

When evaluating PD-1 expression on CD8 T cells (Figure 2B), the frequency of PD-1+ CD8 T cells (median 59.6% in t-BAL and 69.5% in cl-BAL, 32.3% in PB) was significantly higher in t- and cl-BAL than in PB (cl- BAL and t-BAL vs. PB *p* < 0.0001), without significant differences across t- and cl-BAL (*p* = 0.1). As for the mean fluorescence intensity (MFI) of PD-1 on CD8 T cells, this was higher in t- and cl-BAL, despite the difference with PB being significant only for cl-BAL (MFI 29.7 in t-BAL, 34.3 in cl-BAL, 25.3 in PB; t-BAL vs. PB *p* = 0.08, cl-BAL vs. PB *p* = 0.002). The trend for the increased expression of PD-1 on CD8 T cells in t-BAL and cl-BAL compared to PB was confirmed even when considering the groups of NSCLC and squamous cancer patients (see Figure S4).

Even though highly variable across the study population, half or more of the terminally differentiated CD8 T cells in t-BAL and cl-BAL were PD-1+ (median 49.6% in t-BAL, 64.2% in cl-BAL, *p* = 0.5), while in PB the frequency of PD-1+ terminally differentiated CD8 T cells was significantly lower (median 23.1%; PB vs. t-BAL and cl-BAL *p* = 0.03). Similarly, the majority of effector memory CD8 T cells in t- and cl-BAL highly expressed PD-1, as opposed to PB (median 70% in t-BAL, 76.2% in cl-BAL, 41.9% in PB, t-BAL and cl-BAL vs. PB *p* < 0.0001).

To explore the magnitude of exhaustion at the regional level (BAL) compared to the systemic compartment (PB), we assessed on PD-1+ CD8 T cell the co-expression of the costimulatory receptor CD28, which has been shown to characterize early exhausted CD8 T cells with proliferative potential after immunotherapy with PD-1 blockade [18,19]. As shown in Figure 2C, no differences were noted in the early exhausted CD8 T cells across BAL and blood (median 11.1% in t-BAL, 12.7 in cl-BAL, 12.7 in PB), however PD-1+ CD28- CD8 T cells, potentially more exhausted, were enriched in both t-BAL and cl-BAL (median 48.2% in t-BAL vs. 56% in cl-BAL (*p* = 0.2); median 14.6 in PB, cl-BAL and t-BAL vs. PB *p* < 0.0001).

An immune-exhausted microenvironment in BAL from LC patients was further suggested by the reduced ability of CD8 T cells to produce IFN-g after ex vivo stimulation with PMA/Ionomycin/Brefeldin (Figure 2D), compared to PB. Median IFN-g+ CD8 T cells were 23.4% in the t-BAL, 22.9% in c-BAL and 56.4% in PB (t-BAL vs. PB *p* = 0.01; cl-BAL vs. PB *p* = 0.001, t-BAL vs. cl-BAL *p* = 0.7). Consistent with these findings, in t-BAL, PD-1 MFI on CD8 was negatively correlated with T cell ability to produce IFN-g (Figure 2E). In addition, we discriminated t-BAL samples with low vs. high %PD-1+CD28- CD8 T cells based on the median value (47.8% IQR 29–62), and observed that IFN-g was higher in t-BALs with low %PD-1+CD28- CD8 T cells (mean%CD8 producing IFN-g 43.1 vs. 20 in low and high %PD-1+CD28- CD8 T cells, *p* = 0.049), supporting the more exhausted state of this PD-1+CD28- subpopulation of CD8 T cells (Figure 2E).

### 3.4. CD4 T Cells Subsets

**Figure 3:**
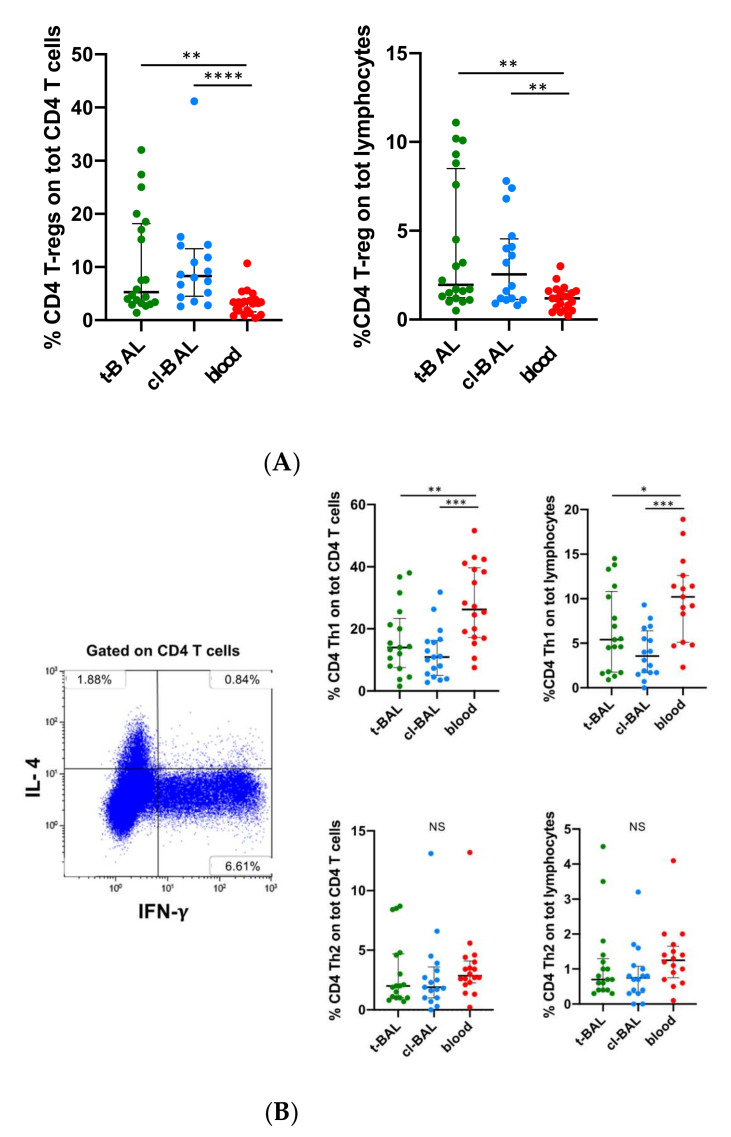

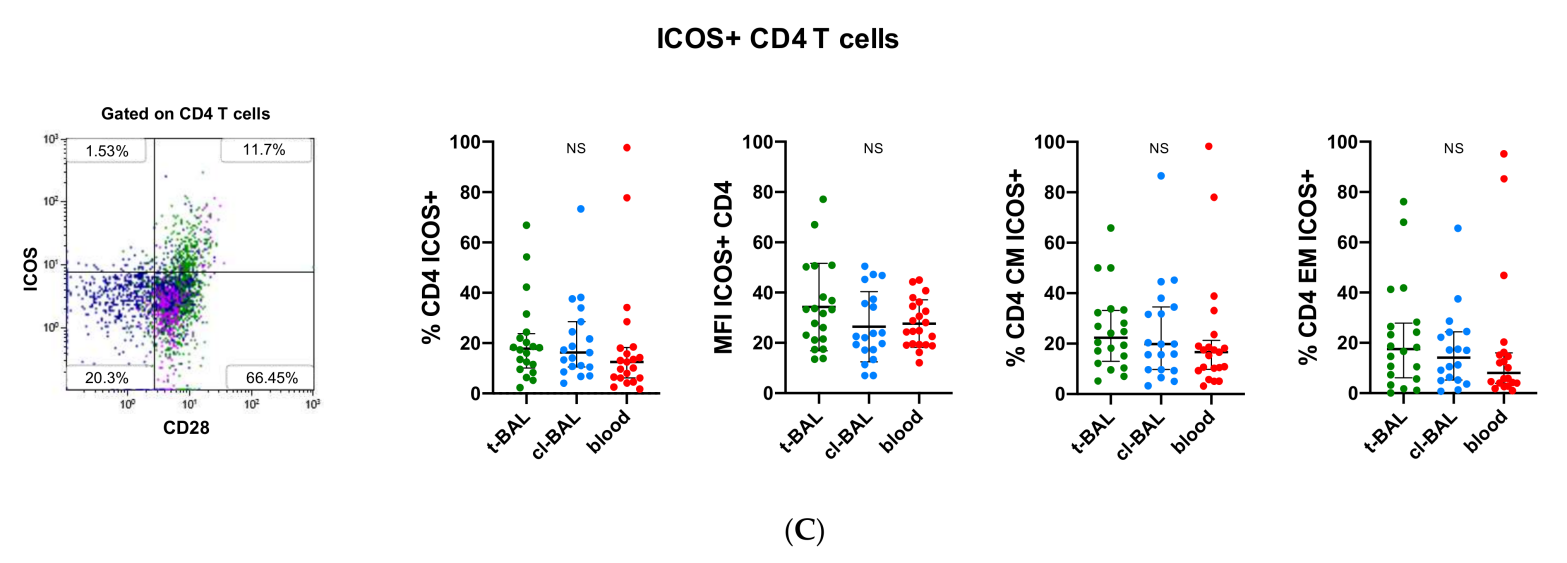
(**A**) Dot plots show the frequencies of T regulatory cells (FOXP3^+^CD25^+^ CD4^+^) on total CD4 and on total lymphocytes, respectively, in t-BAL, cl-BAL, and PB of the cohort (21 patients). (**B**) FACS plot from the t-BAL of a patient showing intracellular interferon-g (IFN-g and IL-4 staining in CD4 T cells. Dot plots showing frequencies of T helper 1 (Th1) CD4 T cells (producing IFN-g on total CD4 T cells and on total lymphocytes, and frequencies of T helper 2 (Th2) CD4 T cells (producing IL-4 on total CD4 T cells and on total lymphocytes in t-BAL, cl-BAL, and PB of the cohort (21 patients) assessed with intracellular cytokine staining with PMA/Ionomycin/Brefeldin. (**C**). FACS plots from the t-BAL of a patient showing ICOS^+^ CD4 T cells (in green central memory, in blue effector memory, in pink naïve CD4 T cells). Dot plots showing, from left to right, frequency of ICOS^+^CD4 T cells on total CD4 T cells, median fluorescence intensity (MFI) of ICOS on CD4 T cells, frequency of ICOS^+^ central memory (CM) C4 T cells on total CM CD4 T cells and frequency of ICOS^+^ effector memory (EM) C4 T cells of total TD CD4 T cells, across t-BAL, cl-BAL, and PB of the cohort (21 patients). Mann–Whitney test was performed to detect statistically significant differences across the groups, except for MFI, where T-test was used. NS: non-significant. *: *p* < 0.05; **: *p* < 0.01; ***: *p* < 0.001; ****: *p* < 0.0001.

Relative %CD4 T cell subsets are shown in Table S3. The exhausted CD8 T cell compartment observed in BAL from LC patients was accompanied by immune-suppressive CD4 T cell characteristics, for expansion of T-regulatory cells (FOXP3+CD25+) (Figure 3A). A significant increase of the T-regulatory cells was observed in both t- and cl-BAL, compared to PB (median frequency 5.2% in t-BAL; 8.3% in cl-BAL; 3.2% in PB, t- vs. cl-BAL *p* = 0.7; t-BAL vs. PB *p* = 0.002, cl-BAL vs. PB *p* < 0.0001). The difference remained significant even when normalizing T-regulatory cells frequency on total lymphocyte count (median frequency 1.95% in t-BAL, 2.5 in cl-BAL, 1.2% in PB, t- vs. cl-BAL *p* = 0.6; t-BAL vs. PB *p* = 0.002, cl-BAL vs. PB *p* = 0.007). Considering T-regs frequencies across t-, cl-BAL, and PB in the NSCLC group, the results were comparable to those observed in the entire study population (the median frequencies of T-regs on total lymphocytes were 1.7% in t-BAL, 1.6% in cl-BAL, 1.2% in PB; t- vs. cl-BAL *p* = 0.4; t-BAL vs. PB *p* = 0.005; cl-BAL vs. PB *p* = 0.03). When considering squamous histology, statistical significance was lost, most likely due to the low numbers per group (11 patients) (1.6% in t-BAL, 1.4% in cl-BAL, 1.2% in PB; t- vs. cl-BAL 0.7; t-BAL vs. PB *p* = 0.1, cl-BAL vs. PB *p* = 0.2) (see Figure S5).

When exploring the CD4 T helper populations, shown in Figure 3B, the Th1 subset, characterized by IFN-g production, was significantly expanded in PB compared to t-and cl-BAL (median frequency 14% in t-BAL, 10.9% in cl-BAL, 26.3% in PB; t- vs. cl-BAL *p* = 0.3, t-BAL vs. PB *p* = 0.006, cl-BAL vs. PB *p* = 0.0001). Comparable results were observed when normalizing the frequency of Th1 CD4 T cells on total lymphocytes (5.4% in t-BAL, 3.5% in cl-BAL, 10.2% in PB; t- vs. cl-BAL *p* = 0.1, t-BAL vs. PB *p* = 0.04, cl-BAL vs. PB *p* = 0.0002).

Relative and normalized frequencies of Th2 and Th17 CD4 T cells (the latter are shown in Figure S6), the first characterized by IL-4 production and the second by IL-17 production, were low, and comparable across t-, cl-BAL, and PB (the median frequencies of Th2 of CD4 T cells were 2% in t-BAL, 1.9% in cl-BAL, and 2.8% in PB. t- vs. cl-BAL *p* = 0.7; t-BAL vs. PB *p* = 0.2; cl-BAL vs. PB *p* = 0.1); median frequencies of Th2 on total lymphocytes were 0.7% in t- and cl-BAL, 1.2% for PB (t- and cl-BAL vs. PB *p* = 0.1); median frequencies of Th17 on CD4 T cells were 1% for t-BAL, 0.9% for cl-BAL, 0.7% for PB (t- vs. cl-BAL *p* = 0.5; t-BAL vs. PB *p* = 0.2; cl-BAL vs. PB *p* = 0.5); median frequency of Th17 on total lymphocytes were 0.3% in t- and BAL, 0.2% in PB (t- BAL vs. PB *p* = 0.1; cl-BAL vs. PB *p* = 0.9).

The ICOS co-stimulatory marker could identify CD4 T follicular helper cells, a specialized subset predominant in the germinal centers of secondary or tertiary lymphoid structures, where they interact with B cells stimulating antibody production or memory B cells differentiation [20]. As shown in Figure 3C, T follicular helper frequency was comparable across t-, cl-BAL, and PB (median 17.8% in t-BAL, 16.3% in cl-BAL and 12.5% in PB; t- vs. cl-BAL *p* = 0.9, t-BAL and cl-BAL vs. PB *p* = 0.1). MFI of ICOS on CD4 T cells was also non-significantly different in t-, cl-BAL, and PB, despite being numerically higher in t-BAL (mean 34.3 in t-BAL, 26.4 in cl-BAL and 27.6 in PB; t-BAL vs. cl-BAL and PB *p* = 0.1, cl-BAL vs. PB *p* = 0.7). ICOS was expressed mostly on central memory (median frequency 22.3% t-BAL, 19.8% cl-BAL, 16.6% in PB, t- vs. cl-BAL *p* = 0.6, t-BAL vs. PB *p* = 0.1, cl-BAL vs. PB *p* = 0.5) and effector memory subsets (median 17.5% in t-BAL, 14.5% in cl-BAL, 8% in PB, t- vs. cl-BAL *p* = 0.6, t-BAL vs. PB *p* = 0.2, cl-BAL vs. PB *p* = 0.3). In these populations, frequencies were highly variable, without significant differences across t-, cl-BAL, and PB, despite showing a trend for increased values in t-BAL.

### 3.5. Clinico-Pathological Associates of T Cell Density in t-BAL

**Figure 4:**
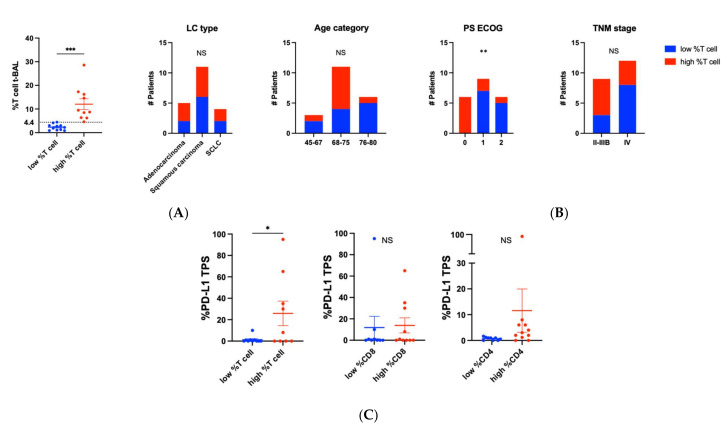
Clinico-pathological associates of T cell density in t-BAL. (**A**) Dot plot showing that low vs. high T cell density in t-BAL was determined based on the median frequency of T cells of all cells in t-BAL (dashed line on the y axis). T-test was used to detect significant differences. (**B**) The distribution of low and high T cell density in t-BAL according to clinical characteristics (lung cancer type, age, ECOG performance, and TNM stage) of the study population. Chi-square test was used to detect significant association between the clinical variable(s) and T cell density. (**C**) PD-L1 tumor proportion score (TPS) on biopsy tissue in low vs. high CD3/CD8/CD4 cell density in t-BAL. T-test was used to detect significant differences. NS: non-significant. *: *p* < 0.05; **: *p* < 0.01; ***: *p* < 0.001.

In t- and cl-BAL, the frequency of T cells and of CD8 and CD4 subsets presented a skewed distribution in the study population. Thus, based on the median value of %T cells in t-BAL of total t-BAL cells (4.4%, IQR 2–9), we dichotomized T cell density in t-BAL into low and high (Figure 4A) and evaluated its distribution across clinico-pathological variables (Figure 4B). Whether T cell density was not related to LC type or age, we found a significant association with ECOG PS, where all t-BAL from patients in optimal clinical conditions (PS = 0) presented high T cell density (Chi-square *p*= 0.02). Early TNM stage (II-IIIB) also tended to be associated with high T cell density, even though the association was not significant (6 of 9 patients with stage II-IIIb had high T cell density in t-BAL, Chi-square *p* = 0.3). Similar clinical associations were observed also for CD8 and CD4 T cell density in t-BAL (Figure S7). Importantly, PD-L1 expression was significantly higher in LC with high T cell density in t-BAL (mean PD-L1 TPS 26% in high %T cell vs. 1.3% in low %T cell, *p* = 0.04) (Figure 4C). PD-L1 expression showed a similar non-significant trend for CD4 T cell density (mean %PD-L1 TPS 0.7 vs. 11.5 in low and high %CD4 T cell, *p* = 0.3), whether PD-L1 expression was comparable in low vs. high CD8 T cell density in t-BAL (mean %PD-L1 TPS 11.9 vs. 13.9 in low and high %CD8 T cell, *p* = 0.87).

### 3.6. Immunological Characteristics in t-BAL with Low vs. High T Cell Density

**Figure 5:**
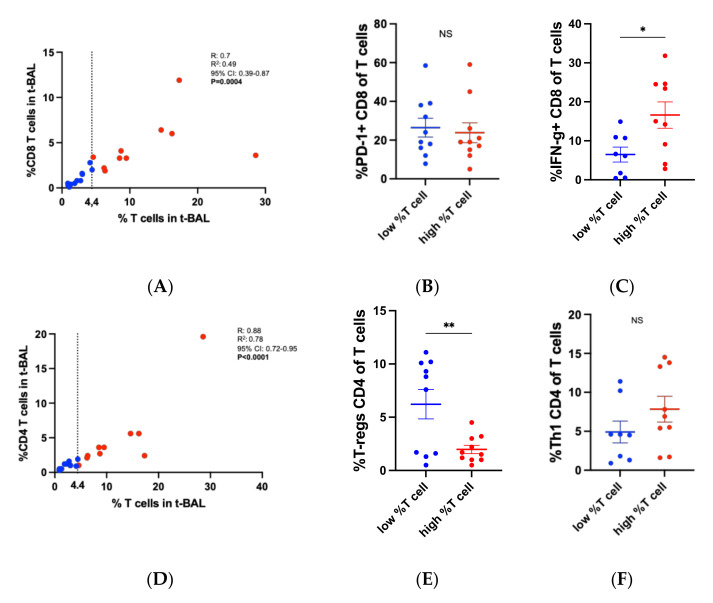
Immunological characteristics in t-BAL with low vs. high T cell density. (**A**) Correlation between %CD8 T cells and %T cells in t-BAL. (**B**) Frequency of PD-1+ CD8 T cell in t-BAL with low vs. high T cell density. (**C**) Frequency of CD8 T cells producing interferon-gamma in t-BAL with low vs. high T cell density. (**D**) Correlation between %CD4 T cells and %T cells in t-BAL. (**E**) Frequency of T-regulatory cells in t-BAL with low vs. high T cell density. (**F**) Frequency of T helper 1 CD4 T cells in t-BAL with low vs. high T cell density. Data were analyzed with T-test, except for (**A**,**D**), where Pearson’s test was used. NS: non-significant. *: *p* < 0.05; **: *p* < 0.01.

T density in t-BAL showed a linear positive correlation with CD8 T cell frequency (Figure 5A), indicating that T cell density in t-BAL can also be a surrogate for CD8 T cell. The frequency of PD-1+ CD8 T cells was comparable in t-BAL with low and high T cell density (Figure 5B). Notably, t-BAL with high T cell density was enriched with IFN-g producing CD8 (mean % 6.5 vs. 16.6 in low vs. high %T cell in t-BAL, *p* = 0.02, Figure 5C).

A positive linear correlation was also found between CD4 and total T cells in t-BAL (Figure 5D). T-regulatory cells were significantly lower in t-BAL with high T cell density (mean % 6.2 vs. 2 in low vs. high %T cell in t-BAL, *p* = 0.008, Figure 5E), whereas no differences were seen for the frequency of Th1 CD4 T cells in low vs. high T cell density t-BAL (mean % 5 vs. 7.8 in low vs. high %T cell, *p* = 0.2, Figure 5F).

## 4. Discussion

In this exploratory descriptive study, we showed the T cell subsets in t- and cl-BAL and how they compare with PB in advanced LC patients. Importantly, we found relevant associations between T cell density in t-BAL and clinico-pathological characteristics, in particular with PDL-L1 TPS. 

Both t- and cl-BAL were enriched in CD8 T cellular immunity, whose predominant phenotype was effector memory. The co-inhibitory receptor PD-1 is a hallmark of T cell exhaustion, induced and maintained by T cell receptor (TCR) stimulation [21]. In our work, the high PD-1 levels observed in effector CD8 T cells, and in particular the inverse correlation between PD-1 MFI and IFN-g production, likely reflect an exhausted immune tumor microenvironment. 

With regard to the CD4 T cell compartment, in both t-and cl-BAL, T-regulatory cells were increased, indicating immune-suppression. The expansion of the T-regulatory cell compartment is in line with the overexpression of the co-inhibitory receptor PD-1 on CD8 T cells, and the increased Th1 functions observed in our study [22]. Indeed, studies have shown that these T cell populations can be regulated by the same cytokines or transcription factors (IFN-g, IL-2, t-bet), in a cross-talk developed to maintain the appropriate homeostasis that prevents self-damage but finally results in T cell exhaustion [23].

Major differences in both CD8 and CD4 T cell populations were not seen among t- and cl-BAL. This could be partially explained by the fact that most of the patients presented advanced or locally advanced disease. However, similar to our case, a previous study by Kwiecien I et al. reported comparable immune characteristics in healthy cl- and t-BAL from early-stage LC patients [24]. In both this and our works, BAL and PB profiling was not performed on healthy subjects as control, and this represented a limitation of our study. An indirect comparison with published data on T cell subsets reveals that CD8 and CD4 populations largely vary, also among healthy subjects, and their frequency is influenced by age and smoking status [25]. With these constrains, it seems that, in smokers, BAL from healthy subjects tends to be enriched in monocytes over lymphocytes compared to non-smokers, reporting a median proportion of lymphocytes (2.3% vs. 11.7% in non-smokers), which is close to that observed in our LC population (about 5% in t- and cl-BAL). BAL from smoker and non-smoker healthy subjects also presents a higher proportion of T cells and CD8 T cells, compared to PB. FOXP3+ CD4 T cells could also be increased in healthy BAL compared to PB [25]. A previous study by Domagała-Kulawik J reported that the proportions of lymphocytes, T cells and CD8 T cells in BAL were similar in LC patients and healthy smoking subjects. Even though a deeper CD8 and CD4 T cell analysis was not performed, the frequency of activated T cells (CD3+HLA-DR+) was lower in LC patients compared to the controls [26].

Bezel P at al. recently used BAL to investigate an LC immune microenvironment focusing on the cytokine milieu, which was compared with a group of healthy subjects and another group of patients affected by other lung diseases [14]. The cytokine composition was not significantly different across these groups, indicating unspecific inflammation, as concluded by the authors. However, interpretation of this data was affected by the dilution of the BAL samples, which was an intrinsic technical limitation. 

In our study, the majority of the immune populations greatly varied within t-BAL samples, therefore we could retrospectively consider T cell density as a dichotomous variable. T cell density in t-BAL resulted as a potential indicator of T cell functionality, and might be influenced by clinico-pathological characteristics. ECOG PS was associated with T cell density, where high T cell density was prevalent in patients in good clinical conditions. On the other hand, t-BAL T cell density was not associated with age or type of LC. Further associations with clinical subgroups (i.e., patients who had received previous systemic treatments or RT at the time of BAL, gender, smoking status) were precluded by the heterogeneity and the small sample size of the study population, which represented, per se, another limitation of our study.

Interestingly, PD-L1 TPS was significantly higher in tumors with high T cell density in t-BAL than in those with low T cell density, suggesting that T cell density may inform on the degree of local immune activation in LC. In support of this hypothesis, t-BAL with high T cell density had an immune composition considerably different from t-BAL with low T cell density, in terms of better cytotoxicity (higher %IFN-g producing CD8 T cells) and lower levels of immune suppression (lower %T-regs).

So far, the available evidence on the correlation of PD-L1 TPS with T cell populations pertains to tumor tissue, mainly examining CD8 T cell infiltration at tumor sites. For ovarian cancer, gastric carcinoma, and cholangiocarcinoma, PD-L1 TPS was positively associated with (CD8) T cells infiltration [27,28,29], but in NSCLC a significant correlation was not found [30].

To our knowledge, this is the first report to explore the clinico-pathological associates of T cell density in t-BAL from LC patients and to compare CD8 and CD4 T cell characteristics in t-BAL with high and low T cell density. Based on our preliminary findings, the T cell density in t-BAL deserves to be prospectively explored in future studies. To confirm its potential value in reflecting the immune activation in an LC microenvironment (inflamed vs. non-inflamed), studies comparing and contrasting T cell phenotype and density in t-BAL and in resected tumor tissue would be needed.

Along the lines of the immunoscore validated for colorectal cancer, it has been recently proposed to prognostically classify NSCLC using a composite evaluation of PD-L1 TPS with CD8 T cell infiltration on a tumor resected specimen [31]. 

In addition, recent studies evaluating T cell populations on tissue specimens have shown that a pre-existing intra-tumor adaptive immune response is essential for an effective response to immunotherapy with checkpoint inhibitors [32,33,34].

In this context, it would be worth to assess if T cell phenotype and density in t-BAL could play a prognostic and/or a predictive role for immunotherapy outcome. 

Notwithstanding the practical issues of repeating BAL sampling in terms of patients’ tolerability, future studies should assess BAL longitudinally, along the different phases of the disease history and treatment outcomes, in order to dissect the complex network regulating responses and toxicity after cancer immunotherapy. The use of novel single-cell transcriptomics and epitope mapping prediction tools may lead to the identification and monitoring of tumor-specific T cells in different stages of activation and exhaustion. For this purpose, it seems likely that BAL could be the most appropriate source to investigate tumor-specific T cells, compared to the heterogeneous T cell repertoire in PB, and considering the poor availability of surgical tissue in clinical practice. Potential findings could clarify the T cell effects of cancer immunotherapy and set the basis for the design of treatment strategies to enhance or suppress the specific T cell populations involved in anti-cancer response.

## Figures and Tables

**Table 1:** Antibody mixtures, fluorochromes (PB: Pacific Blu, KO: Krome Orange) and antibody sources (BC: Beckman Coulter, MY: Miltenyi Biotech, eBio: eBioscience).

	Fluorochrome								
	PB	KO	FITC	PE	PerCP	PC5.5	PC7	APC	APC-AF750
Mix 1 BAL basic phenotype	CD8 (BC)	CD45 (BC)	CD57 (BC)	CD19 (BC)			CD4 (BC)	CD56 (BC)	CD3 (BC)
Mix 2 PB basic phenotype	HLA-DR (BC)	CD45 (BC)	CD8 + 20 (BC)	CD16 + 56 (BC)		CD5 + 14 (BC)	CD19 (BC)	CD4 (BC)	CD3 (BC)
Mix 3 T-cell subsetting	CD27 (BC)	CD8 (BC)	CD4 (BC)	CD278 (BC)	CD3 (MY)		CD279 (BC)	CD45RA (BC)	CD28 (BC)
Mix 4 Treg phenotype	FoxP3 (eBio)		CD3 (BC)	CD4 (BC)			CD127 (BC)	CD25 (BC)	CD45RA (BC)
Mix 5 Th1, Th2, Th17	a-IL17 (eBio)	CD45 (BC)	a-IFNg (eBio)				a-IL4 (eBio)	CD4 (BC)	CD3 (BC)

**Table 2:** General characteristics of the study population.

Patients’ Characteristics		N (%) Tot = 21 Patients
Median age (years)		73 (range 45–80)
Gender (male)		17 (81)
Smokers/former smokers		20 (95)
ECOG PS	0–1	16 (76)
	2	5 (24)
Small-cell histology		4 (19)
Non-small cell histology	Adenocarcinoma	6 (28.6)
	Squamous cell	11 (52.4)
Median PD-L1 (TPS score %)		0.5 (0–25)
TNM stage at BAL *	II	2 (9.5)
	III	7 (33.3)
	IV	12 (57.1)
Previous treatments	Surgery **	3 (14.2)
	Chemotherapy **	3 (14.2)
	Tyrosine-kinase inhibitor **	1 (4.8)
	RT **	2 (9.5)
	None	17 (80.9)
Treatment after BAL	Surgery ^§^	2 (9.5)
	Chemo-RT ^§^	5 (23.8)
	Chemotherapy ^§^	10 (47.6)
	Immunotherapy ^§^	4 (19)
	Tyrosine-kinase inhibitor ^§^	1 (4.8)

* Inclusive of both NSCLC and SCLC (8th edition). ** One patient received adjuvant chemotherapy and later gefitinib. One patient received sequential chemo-radiotherapy. One patient underwent radical lobectomy. One patient underwent radical lobectomy and more than 5 years later received thoracic radiotherapy for tumor recurrence. Due to disease progression 2 years later this same patient was treated with platinum-based chemotherapy. ^§^ After BAL, one patient received chemo-radiotherapy and for progression 2nd line pembrolizumab; 2 patients received concomitant chemo-radiotherapy followed by durvalumab maintenance. Abbreviations: ECOG PS, Eastern Cooperative Oncology Group Performance Status, TPS, tumor proportion score, RT, radiotherapy.

## Data Availability

Not applicable.

## Supplementary Materials

The following supporting information can be downloaded at: https://www.mdpi.com/article/10.3390/cells11203226/s1, Figure S1: BAL gating strategy to identify and isolate lymphocyte population. Figure S2: Peripheral blood gating strategy to identify and isolate lymphocyte population. Figure S3: Dot plot showing frequency of EM of CD8 T cells across t-BAL, cl-BAL and PB in the groups of NSCLC patients (A) and squamous LC patients (B). Figure S4. Dot plot showing median fluoresce intensity (MFI) of CD8 T cells across t-BAL, cl-BAL and PB in the groups of NSCLC patients (A) and squamous LC patients (B). Figure S5. Dot plot showing frequency of T-regulatory cells (FOXP3+CD25+CD4+) of total lymphocytes in t-BAL, cl-BAL and PB in the groups of NSCLC (A) and squamous cancer patients (B). Figure S6. Dot plot showing frequency of Th17 CD4 T cells of total CD4 T cells (A) and of total lymphocytes (B) in t-BAL, cl-BAL and PB. Figure S7. Dot plot showing that low vs high CD8 T cell density in t-BAL was determined based on its median frequency of all cells in t-BAL (1.95% IQR 0.65–3.5, dashed line on the y axis); Table S1: Relative % (on parent population) of immune cells in t-BAL, cl-BAL and PB. Table S2. Median % of CD8 T cell subsets. Table S3. Relative %CD4 T cell subsets.
