# Supplementary material for: Comparing T Cell Subsets in Broncho-Alveolar Lavage (BAL) and Peripheral Blood in Patients with Advanced Lung Cancer
Source: Cells. 2022 Oct 14;11(20):3226. doi: 10.3390/cells11203226 (PMC9600421; doi:10.3390/cells11203226)

Figure S1. BAL gating strategy to identify and isolate lymphocyte population. Plot A: doublet exclusion gate (P). Plot B: debris exclusion gate (O). Plot C: lymphocyte gate (in red).

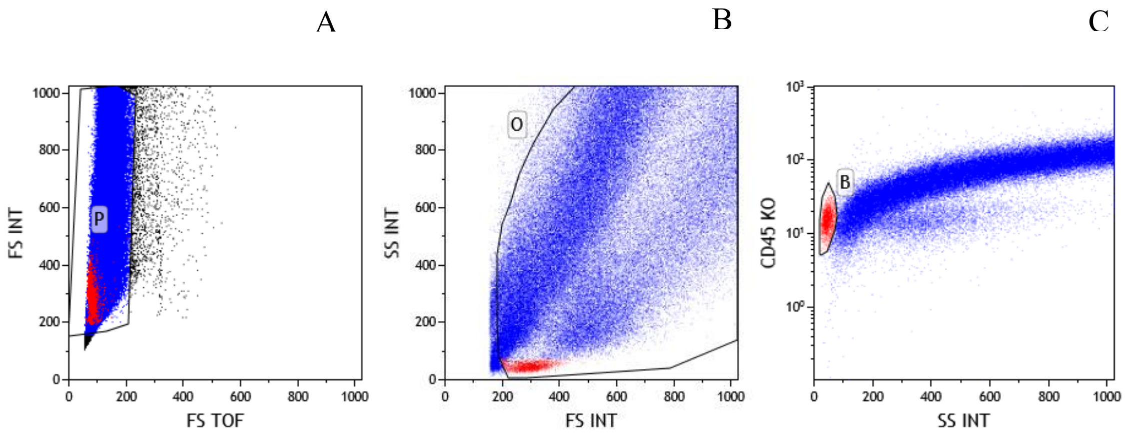

Figure S2. Peripheral blood gating strategy to identify and isolate lymphocyte population. Plot A: doublets exclusion gate. Plot B: leucocytes and debris gates. Plots C and D: lymphocytes isolation gates (A and C)

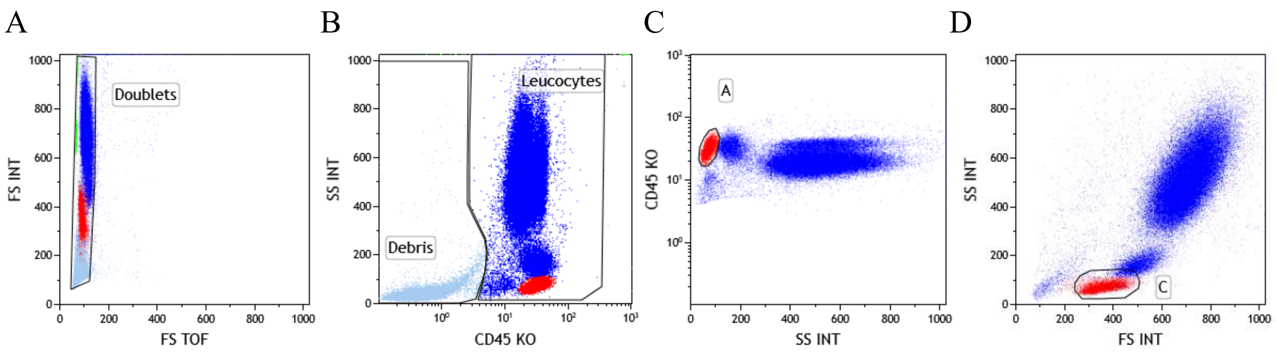

Table S1. Relative % (on parent population) of immune cells in t-BAL, cl-BAL and PB.

		t-BAL	cl-BAL	PB	Mann-Whitney test
Lymph. %	Median (Range)	4.8 (0.9-35.2)	5.4 (0.6-32.9)	21.0 (10-38)	t-BAL vs PB p<0.0001<br>cl-BAL vs PB p<0.0001<br>t-BAL vs cl-BAL p=0.79
T Lymph. (CD3+)	Median (Range)	83.4 (46.8-96.1)	88.8 (49-96.1)	72.8 (47.5-86.3)	t-BAL vs PB p<0.001<br>cl-BAL vs PB p<0.001<br>t-BAL vs cl-BAL p=0.56
CD4 T Lymph. (CD3+CD4+)	Median (Range)	37.6 (14-68.7)	29.3 (13-63.8)	42.2 (22-67.7)	t-BAL vs PB p=0.71<br>cl-BAL vs PB p=0.04<br>t-BAL vs cl-BAL p=0.08

CD8 T Lymph. ($CD3^+CD8^+$)	Median (Range)	37.2 (12.1-74.5)	48.6 (20.5-82)	27.0 (8.5-56.3)	t-BAL vs PB p=0.02<br>cl-BAL vs PB p<0.0001<br>t-BAL vs cl-BAL p=0.06
NK Lymph. ($CD3^-CD16^+56^+$)	Median (Range)	5.8 0.9-25.5	3.2 0.4-32.6	15.7 5-40.4	t-BAL vs PB p<0.001<br>cl-BAL vs PB p=0.001<br>t-BAL vs cl-BAL p=0.3
B Lymph. ($CD19^+$)	Median (Range)	3.9 (0.4-42.7)	2.3 (0.2-21.6)	8.5 (2.9-26.7)	t-BAL vs PB p<0.001<br>cl-BAL vs PB p<0.0001<br>t-BAL vs cl-BAL p=0.2

Table S2. Median % of CD8 T cell subsets.

			t-BAL	cl-BAL	PB	Mann-Whitney test
CD8	Naive	Median (Range)	14.7 (0.3-38.7)	4.2 (0.5-31.1)	33.2 (3.5-85.4)	t-BAL vs PB p=0.01<br>cl-BAL vs PB p<0.0001<br>t-BAL vs cl-BAL p=0.16
	Central Memory	Median (Range)	26.4 (3.2-54.3)	15.7 (2.6-70.7)	24.9 (3.5-74.4)	t-BAL vs PB p=0.7<br>cl-BAL vs PB p=0.4<br>t-BAL vs cl-BAL p=0.6
	Effector Memory	Median (Range)	54.1 (23-96)	63.1 (22.2-94.2)	17.5 (2.3-60.4)	t-BAL vs PB p<0.0001<br>cl-BAL vs PB p<0.0001<br>t-BAL vs cl-BAL p=0.3
	Terminally differentiated	Median (Range)	5.1 (0-18.3)	1.6 (0-47.3)	12.7 (1.4-47.9)	t-BAL vs PB p=0.003<br>cl-BAL vs PB p<0.001<br>t-BAL vs cl-BAL p=0.06
	PD-1+	Median (Range)	59.6 (27.5-89.9)	69.5 (40.8-91)	32.3 (12.4-56.5)	t-BAL vs PB p<0.0001<br>cl-BAL vs PB p<0.0001<br>t-BAL vs cl-BAL p=0.1
	PD-1+ CD28+	Median (Range)	12.5 (3.6-8.6)	12.75 (1-45.7)	13.35 (3.1-48.1)	t-BAL vs PB p=0.67<br>cl-BAL vs PB p=0.25<br>t-BAL vs cl-BAL p=0.34

	PD-1+ CD28-	Median (Range)	47.8 (15.9-78.6)	56 (27.1-89.3)	14.45 (1.9-32.7)	t-BAL vs PB p<0.0001<br>cl-BAL vs PB p<0.0001<br>t-BAL vs cl-BAL p=0.13
	IFN-g+	Median (Range)	23.4 (1.8-71)	22.9 (2.5-57.8)	56.4 (1.7-83.5)	t-BAL vs PB p=0.01<br>cl-BAL vs PB p=0.001<br>t-BAL vs cl-BAL p=0.7
EM CD8	PD-1+	Median (Range)	70.0 (23.4-95.8)	76.2 (39.5-96)	41.9 (4.8-65.3)	t-BAL vs PB p<0.0001<br>cl-BAL vs PB p<0.0001<br>t-BAL vs cl-BAL p=0.6
TD CD8	PD-1+	Median (Range)	49.6 (16-82.4)	64.2 (12.3-90.9)	23.1 (4.3-81.6)	t-BAL vs PB p=0.03<br>cl-BAL vs PB p=0.03<br>t-BAL vs cl-BAL p=0.5

Figure S3. Dot plot showing frequency of EM of CD8 T cells across t-BAL, cl-BAL and PB in the groups of NSCLC patients (A) and squamous LC patients (B). *: P<0.05; **: P<0.01; ***: P<0.001; ****: P<0.0001.

A B

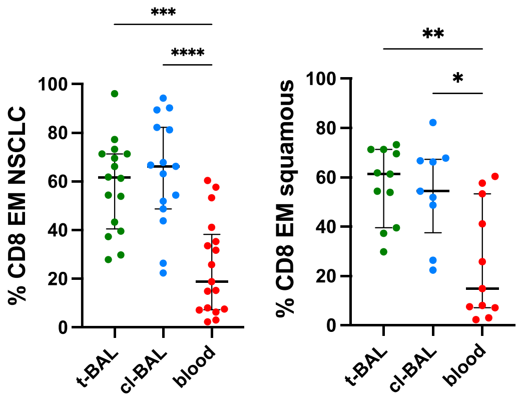

Figure S4. Dot plot showing median fluoresce intensity (MFI) of CD8 T cells across t-BAL, cl-BAL and PB in the groups of NSCLC patients (A) and squamous LC patients (B). *: P<0.05; **: P<0.01; ***: P<0.001; ****: P<0.0001.

A B

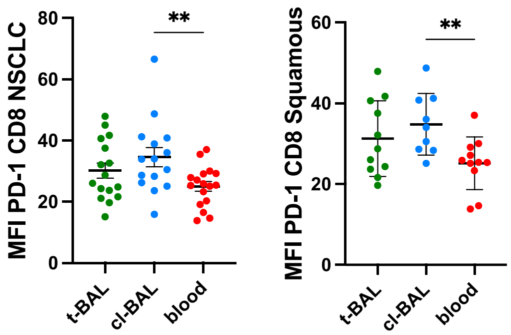

Table S3. Relative %CD4 T cell subsets.

			t-BAL	cl-BAL	PB	Mann-Whitney test
CD4	T-regulatory	Median (Range)	5.25 (1.4-32)	8.3 (2.6-41.2)	3.2 (0.4-10.7)	t-BAL vs PB p=0.002<br>cl-BAL vs PB p<0.0001<br>t-BAL vs cl-BAL p=0.67
	Th1	Median (Range)	14 (1.5-38)	10.9 (2.8-31.8)	26.3 (7.5-51.6)	t-BAL vs PB p=0.006<br>cl-BAL vs PB p=0.001<br>t-BAL vs cl-BAL p=0.33
	Th2	Median (Range)	2 (0.7-8.7)	1.9 (0-13.1)	2.8 (0.2-13.2)	t-BAL vs PB p=0.2<br>cl-BAL vs PB p=0.1<br>t-BAL vs cl-BAL p=0.78
	Th17	Median (Range)	1 (0.1-8.7)	0.9 (0-8.5)	0.7 (0.1-2.5)	t-BAL vs PB p=0.003<br>cl-BAL vs PB p<0.001<br>t-BAL vs cl-BAL p=0.06
	ICOS+	Median (Range)	17.8 (2.4-66.9)	16.3 (4.1-73.4)	12.5 (1.8-97.7)	t-BAL vs PB p=0.1<br>cl-BAL vs PB p=0.1<br>t-BAL vs cl-BAL p=0.9
CM CD4	ICOS+	Median (Range)	22.3 (5.2-66)	19.8 (3.3-86.5)	16.6 (3.2-98.3)	t-BAL vs PB p=0.1<br>cl-BAL vs PB p=0.5<br>t-BAL vs cl-BAL p=0.6
EM CD4	ICOS+	Median (Range)	17.5 (0.1-76.2)	14.15 (0.8-65.6)	8 (1-95.2)	t-BAL vs PB p=0.03<br>cl-BAL vs PB p=0.03<br>t-BAL vs cl-BAL p=0.5

Figure S5. Dot plot showing frequency of T-regulatory cells (FOXP3+CD25+CD4+) of total lymphocytes in t-BAL, cl-BAL and PB in the groups of NSCLC (A) and squamous cancer patients (B). *: P<0.05; **: P<0.01; ***: P<0.001; ****: P<0.0001.

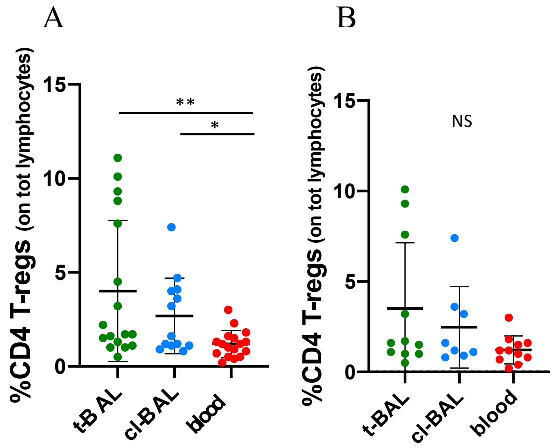

Figure S6. Dot plot showing frequency of Th17 CD4 T cells of total CD4 T cells (A) and of total lymphocytes (B) in t-BAL, cl-BAL and PB . Th17 CD4 T cell were characterized by production of IL-17 after stimulation ex vivo with PMA/Ionomycin/Brefeldin. *: P<0.05; **: P<0.01; ***: P<0.001; ****: P<0.0001.

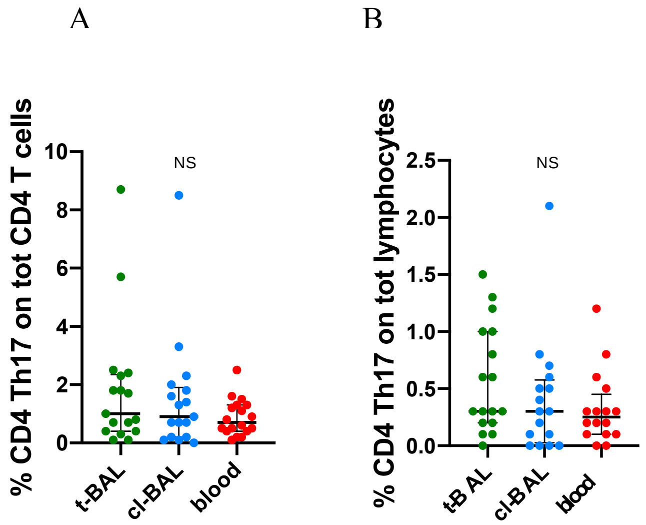

Figure S7. Dot plot showing that low vs high CD8 T cell density in t-BAL was determined based on its median frequency of all cells in t-BAL (1.95% IQR 0.65-3.5, dashed line on the y axis). B) Distribution of low and high CD8 T cell density in t-BAL according to clinical characteristics (lung cancer type, age, ECOG performance and TNM stage) of the study population. C) Dot plot showing that low vs high CD4 T cell density in t-BAL was determined based on its median frequency of all

cells in t-BAL (1.65% IQR 0.95-3.15, dashed line on the y axis). D) Distribution of low and high CD4 T cell density in t-BAL according to clinical characteristics (lung cancer type, age, ECOG performance and TNM stage) of the study population. T-test was used to detect significant differences in A) and C), Chi-square test was used to detect significant association between the clinical variable(s) and CD8 or and CD4 T cell density in B) and D) respectively. NS: non-significant. *: P<0.05; **: P<0.01; ***: P<0.001; ****: P<0.0001.

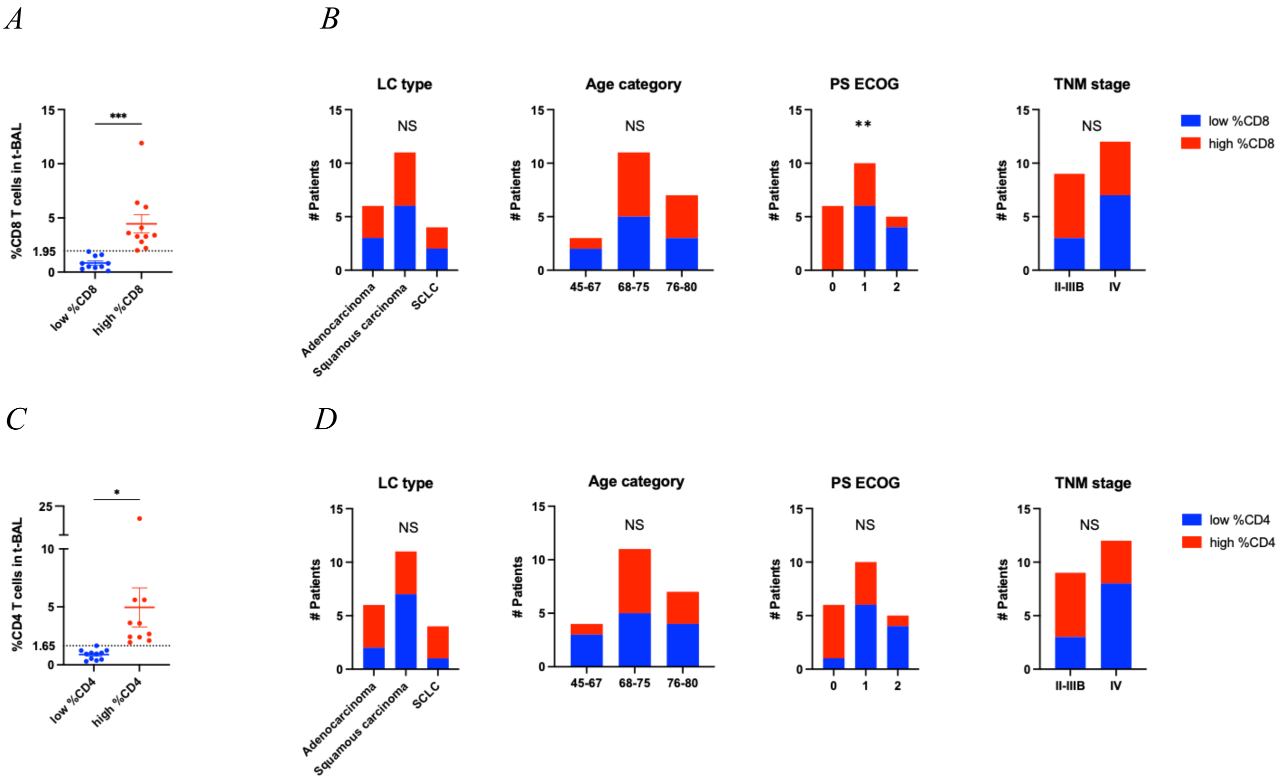

Supplement: Supplementary file 1 [file cells-11-03226-s001.zip › cells-1887151-supplementary.pdf]
